# Monocyte programming by cancer therapy

**DOI:** 10.3389/fimmu.2022.994319

**Published:** 2022-10-20

**Authors:** Marina Patysheva, Anastasia Frolova, Irina Larionova, Sergey Afanas'ev, Anna Tarasova, Nadezhda Cherdyntseva, Julia Kzhyshkowska

**Affiliations:** ^1^ Laboratory of Translational Cellular and Molecular Biomedicine, Tomsk State University, Tomsk, Russia; ^2^ Laboratory of Tumor Progression Biology, Cancer Research Institute, Tomsk National Research Medical Center, Russian Academy of Sciences, Tomsk, Russia; ^3^ Laboratory of Molecular Oncology and Immunology, Cancer Research Institute, Tomsk National Research Medical Center, Russian Academy of Sciences, Tomsk, Russia; ^4^ Laboratory of Genetic Technologies, Siberian State Medical University, Tomsk, Russia; ^5^ Department of Abdominal Oncology, Cancer Research Institute, Tomsk National Research Medical Center, Russian Academy of Sciences, Tomsk, Russia; ^6^ Institute of Transfusion Medicine and Immunology, Institute for Innate Immunoscience (MI3), Medical Faculty Mannheim, University of Heidelberg, Mannheim, Germany; ^7^ German Red Cross Blood Service Baden-Württemberg – Hessen, Mannheim, Germany

**Keywords:** monocyte, anti-cancer treatment, surgery, chemotherapy, radiotherapy, immunotherapy, genotype

## Abstract

Monocytes in peripheral blood circulation are the precursor of essential cells that control tumor progression, that include tumor-associated macrophages (TAMs), dendritic cells (DCs) and myeloid-derive suppressor cells (MDSC). Monocytes-derived cells orchestrate immune reactions in tumor microenvironment that control disease outcome and efficiency of cancer therapy. Four major types of anti-cancer therapy, surgery, radiotherapy, chemotherapy, and most recent immunotherapy, affect tumor-associated macrophage (TAM) polarization and functions. TAMs can also decrease the efficiency of therapy in a tumor-specific way. Monocytes is a major source of TAMs, and are recruited to tumor mass from the blood circulation. However, the mechanisms of monocyte programming in circulation by different therapeutic onsets are only emerging. In our review, we present the state-of-the art about the effects of anti-cancer therapy on monocyte progenitors and their dedifferentiation, on the content of monocyte subpopulations and their transcriptional programs in the circulation, on their recruitment into tumor mass and their potential to give origin for TAMs in tumor-specific microenvironment. We have also summarized very limited available knowledge about genetics that can affect monocyte interaction with cancer therapy, and highlighted the perspectives for the therapeutic targeting of circulating monocytes in cancer patients. We summarized the knowledge about the mediators that affect monocytes fate in all four types of therapies, and we highlighted the perspectives for targeting monocytes to develop combined and minimally invasive anti-cancer therapeutic approaches.

## Introduction

The immune systems can both cooperate with cancer therapy as well as interfere with its effects (1). Monocytes give origin to tumor-associated macrophages (TAM), dendritic cells (DC), and myeloid-derived suppressor cells (MDSC) that control immune reactions and cancer cell biology in tumor microenvironment (TME) (2–4). The functions of these cell types are controlled on the transcriptional, epigenetic, and metabolic levels (5). The differentiation of monocytes after their migration into tissues affects their descendants function and significantly affects efficiency of adaptive immune response, intramural immune status, level of angiogenesis, proliferation of cancer cells (6–9). TAMs are the most diverse and multifunctional orchestrators of cell-cell and cell-matrix interactions in TME. TAMs are programmed on the transcriptional, epigenetic and metabolic levels by multiple factors released by cancer cells, endothelial cells, fibroblasts, innate and adaptive immune cells in TME (5). In the majority of cancers, including breast, lung, prostate, and ovarian cancer, TAM substantially support tumor progression (6). TAMs secreted multiple angiogenic regulators that belong to growth factor family, S100 protein family, soluble ECM regulators, chitinase like proteins and others (10). TAMs can respond to and process number of chemotherapeutic agents, and significantly impair their effects on cancer cells (11). DCs are the main antigen-presenting cells for induction of adaptive immune responses (12). Monocytes can meet cancer-related circulating factors during the time they spend in blood, and can acquire detrimental for the patient programs transferred to tumor tissue and affecting differentiation of TAMs, DCs and MDSCs (13–15). Our understanding of monocytes has advanced when scientist started to consider these cells not as a homogenous population but as heterogeneous system that display responses to different stimuli (7, 16, 17). In cancer, monocyte subsets possess diverse activities that contribute to both pro‐ and anti-tumoral immune response, including phagocytosis, secretion of tumoricidal mediators, angiogenesis, remodeling of the extracellular matrix, recruitment of lymphocytes, as well as differentiation into TAM, MDSCs and DCs (2, 7–9, 18–21).

Human monocytes are a highly heterogenic population of cells, which can be defined by various markers, such as HLA-DR, CX3CR1, CCR2, CCR7, CD62L, Tie2, CD33, CD86, CD141, CD206, and others (22–24). The diversity of monocytes was firstly reflected in the nomenclature developed in the 1980s using 2‐color flow cytometric detection of two universal markers, CD14 and CD16, on human PBMCs (25–27). It allowed to identify 3 subpopulations – classical (CD14++16-), intermediate (CD14+16+) and non-classical (CD14+16++) monocytes (28–30). Later, other functional molecules were found that helped to identify further specific subpopulations of monocytes (**Table 1**). To date, the molecular and functional characteristics of monocyte subpopulations that give origin specifically to TAMs, DCs and/or MDSCs in tumor tissues remains to be unclear.

**Table 1:** Clinical analysis of circulation monocyte diversity in cancer.

Method of detection	Localization	Parameter	Correlation with clinical parameters	Reference
Absolute complete blood count identified by routine laboratory measurements with automated hematology analyzers	Oral cancer	Monocyte number with NLR	Negative correlation with OS	(31)
	Nasopharyngeal carcinoma	LMR	Positive correlation with OS	(32)
	Oesophageal cancer	LMR	Negative correlation with OS	(33)
	Gastric cancer	Low LMR	Predicts poor OS	(34, 35)
	Gallbladder cancer	MLR	High MLR correlates with shorter PFS	(36)
	Ovarian cancer	MLR	Predictor of advanced stages, advanced pathologic grades, and positive lymphatic metastasis	(37)
	Ovarian cancer	LMR	Low LMR was associated with both poor OS, G2/G3 histological grade, III-IV FIGO stage tumors, high serum CA-125 level, presence of malignant ascites and lymph node metastases	(38)
	Prostate cancer	MLR+ monocyte number	Associated with high Gleason score prostate cancer	(39)
	Breast cancer	LMR	Low LMR is considered a favorable predicative factor for NAC efficacy	(40)
	Breast cancer	high LMR	Associated with a lower percentage of relapse in patients treated with NAC	(41)
	Non-small-cell lung cancer	high LMR	Associated with the effects of nivolumab therapy	(42)
	Urothelial cancer	elevated preoperative MLR	Indicated unfavorable OS	(43)
Monocytes subsets in blood identified by flow cytometry	Lung cancer	CD14+CD16+ cells (detected by FACS Canto II, BD Biosciences)	Higher proportion of classical and intermediate monocytes are associated with cancer	(44)
	Colorectal cancer	Tie2+ cells (detected by FC500 or Gallios cytometer, Beckman Coulter)	Increased in patients cohort compared to healthy volunteers	(45)
	Breast cancer	CD14+CD163+ and CD14+CD204+ cells (detected by FACS Navios, Beckman Coulter)	Increased in patients cohort compared to healthy volunteers	(46)
	Prostate cancer	CD14+HLA-DRlow/- cells (detected by FACSCalibur, BD Biosciences)	Increased in patients cohort compared to healthy volunteers	(47)
	Endometrial cancer	CD14, CD16, CD45RA, CD54 (ICAM-1), CD86, HLAI, VEGF-R1 (detected by FACS Canto, BD Biosciences)	Reduced MFI level of CD86, HLA-I, CD45RA, CD54 and up-regulation of VEGF-R1 are associated with cancer	(48)
Transcriptome of circulation monocytes detected by:				
1.Microarray	Human renal cell carcinoma	Specific up-regulated gene signature; IL1b in CD14+ cells (Illumina Human HT-12 v4 Beadchip, BeadArray Scanner 500GX)	Up-regulated in patients cohort compared to healthy volunteers	(49)
	Colorectal cancer	Specific up-regulated gene signature in CD14+ cells (Illumina Human HT-12 v4 Beadchip, BeadArray Scanner 500GX)	Up-regulated in patients cohort compared to healthy volunteers	(15)
2. Bulk RNA-seq	Breast and endometrial cancer	Specific gene signature in CD14+CD16- cells (Ovation RNA-seq Amplification kit v2, Nugen; sequencing were performed by HiSeq 2000 and 2005, Illumina)	Up-regulated in patients cohort compared to healthy volunteers	(14)
	Breast cancer	Specific gene signature in CD14+ cells (NEXTflex Rapid Directional qRNA-Seq Kit, PerkinElmer Applied Genomics, sequencing were performed by NextSeq 550 Illumina)	*CD163 up-regulated* with increase of CD14-16+163+, CD14+16+163+ in patients cohorts compared to healthy volunteers	(50)
3. Single-cell RNA-seq	Lung cancer	Specific clusters and up-regulated gene signature in CD14+CD16- cells (inDrops droplet microfluidics)	Up-regulated in patients cohort compared to healthy volunteers	(51)
	Pancreatic ductal adenocarcinoma	C1QA, C1QB, and TREM2 identified in monocytes populations in PBMC	Monocyte subpopulation with up-regulated *C1QA, C1QB*, and *TREM2*	(52)
Time-of-flight mass cytometry (CyTOF)	Pancreatic ductal adenocarcinoma	The neonatal Fc receptor on monocytic and granulocytic myeloid-derived suppressor cell	The neonatal Fc receptor decreased in monocytic and granulocytic myeloid-derived suppressor cell in patients cohort	(53)
	Colorectal cancer	Circulation immune landscape	In patient cohort CD16- monocytes were increased, but CD16+ monocytes were decreased	(54)

DFS, disease-free survival; LMR, lymphoсyte-to-monocyte ratio; MLR, monocyte-to- lymphocyte ratio; NLR, neutrophil-to-lymphoсyte ratio; OS, overall survival; PFS, progression-free survival; PBMC, peripheral blood mononuclear cell.

Multilevel molecular interactions control monocyte functions (55). Genetic alterations can affect pro- or anticancer potential of monocytes. Genetic polymorphisms of genes coding key cytokines and chemokines, such as factor for monocyte differentiation (macrophage colony-stimulating factor, M-CSF), factors for monocyte recruitment (monocyte chemoattractant protein-1 (MCP-1 or CCL2), CCR5 and CXCR4), are associated with breast, endometrial, oral, lung, prostate and bladder cancers (56–62).

As critical parameters of inflammation, amounts of monocytes or lymphocyte-monocytes ratio (LMR), and content of circulating monocytes subpopulations compositions can have diagnostic and prognostic significance (summarized in **Table 1**) (31, 32, 36–49, 63–68). Unambiguously attractive is the fact that monocytes circulate in blood that makes them minimally invasive biomarkers and accessible cell targets for therapy. Identification of critical molecular mechanisms regulating monocyte programs is needed to find new therapeutic targets. Recent studies using high throughput methods showed that transcriptional alterations in peripheral blood monocytes can serve as diagnostic, predictive and prognostic biomarkers in renal, colorectal, breast, cervical, skin, thyroid, hepatocellular and lung cancers (summarized in **Table 1**) (14, 15, 21, 49, 50, 69, 70).

## Effects of treatment on circulating monocytes

The main goal of antitumor therapy is to achieve the maximal cytoreduction of primary tumor and metastatic foci (71). The increase of anti-tumor treatment efficiency is a major challenge in oncology. Drug resistance, related to tumor cell activity, is the main obstacle leading to inability to achieve pathological complete response (pCR). However, the outcome of cytostatic treatment significantly depends not only on the biological characteristics of tumor cells, but also on their interaction with components of TME (11, 72).

Similar to tumor cells, cells of immune system, which have a high proliferative potential, are also adversely affected by cytostatic treatment. Leukopenia and immunosuppression frequently occur after completion of anticancer treatment. However, there are also many reports indicating immunomodulatory effects of cytotoxic drugs (73–75). Under certain conditions, radiation and drug therapy can diminish tumor escape from immune surveillance, and restore anti-tumor immune response, resulting in increasing therapeutic efficiency (73–76). Several lines of evidences demonstrate that TAMs are accumulated in tumors after chemo- and radiotherapy and contribute to tumor recurrence inducing suppression of T cell immunity, the maintenance of tumor cell survival and activation of tumor vascularization (11). Moreover, TAMs often accumulate in hypoxic regions of solid tumors, since hypoxia mediates recruitment of monocytes and pro-tumor polarization of monocyte-derived cells, including TAMs (64, 77, 78). Thus, hypoxia-inducible factor 1α (HIF-1) directly regulates the function and differentiation of MDSCs in hypoxic zones (79). Monocytes, the main precursors of TAMs, DCs, and MDSCs are programmed by treatment in circulation and the TME. This fact can be used for the predicting anti-tumor therapeutic response and tumor-directed targeting (73–76). Immunotherapy is most recent cancer treatment that has shown good results for solid tumors with reduced side effects compared to conventional treatments (80). However, not all tumors sufficiently respond to immunotherapy (81). Current problem is to identify the criteria that predict efficiency of immunotherapy. Considering involvement of monocytes in tumor pathogenesis, the functional profile of monocytes can be an informative factor for immunotherapy outcome.

Below we summarize accumulated data, indicating how anti-cancer treatment alters monocyte programming in bone marrow and peripheral blood, as well as during the monocyte recruitment to tumor and differentiation towards TAMs, DCs, and MDSCs. We present the state of the art in our knowledge about the mediators that program monocytes during various types of cancer therapy. We highlight the necessity for deep investigation of molecular mechanisms of such programming in order to develop new anti-cancer treatment approaches and to enhance the efficiency of the existing approaches.

### Surgical treatment

**Figure 1:**
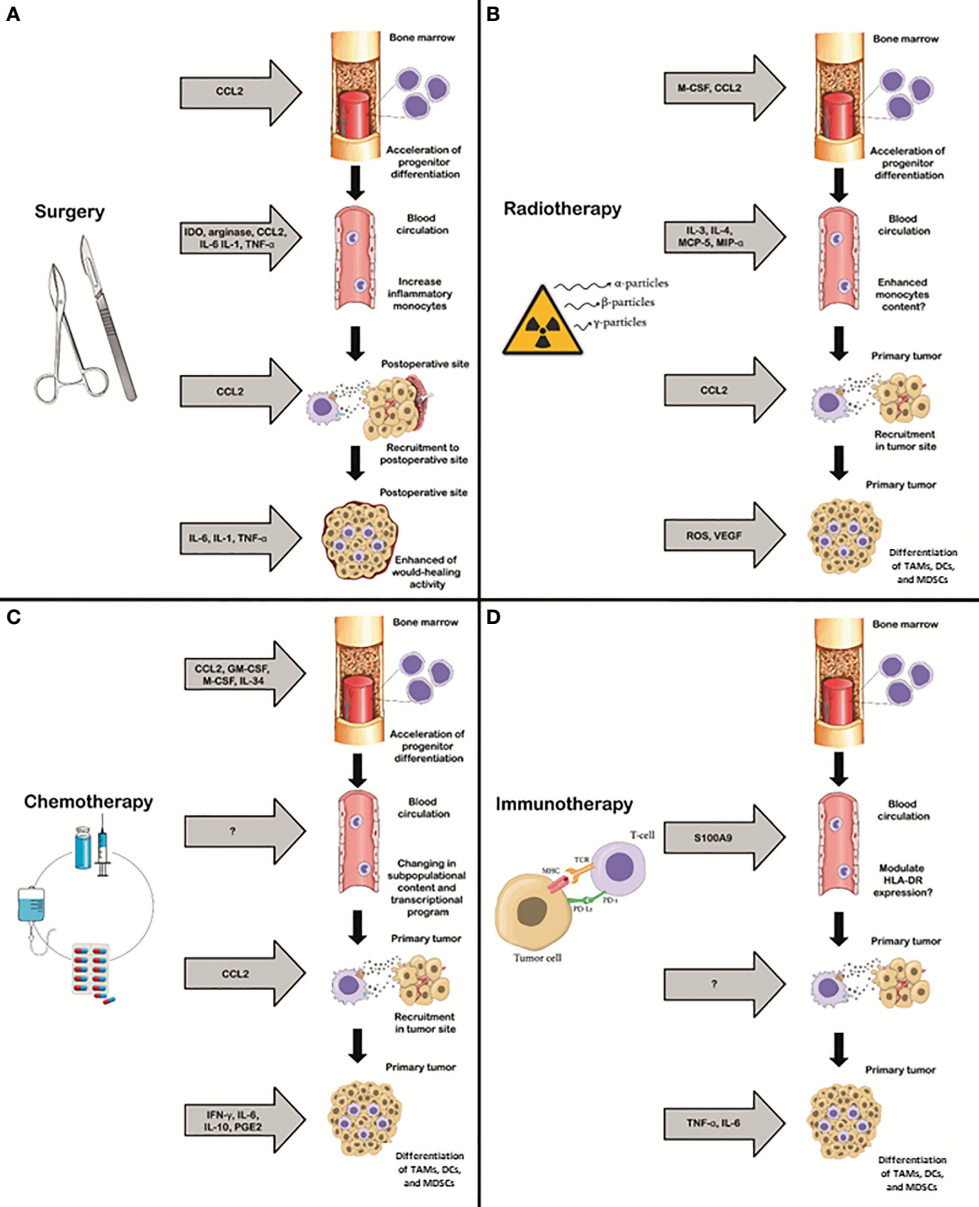
Interaction of four major types of cancer therapy with monocyte programming. Effects of surgery **(A)**, radiotherapy **(B)**, chemotherapy **(C)** and immunotherapy **(D)** on the differentiation of the monocyte progenitors in bone marrow, monocyte amounts and content in the circulation, recruitment to tumor and differentiation into TAMs are separately presented. Soluble mediators of the therapy effects are listed in the grey arrows. GM-CSF- Granulocyte-macrophage colony-stimulating factor, M-CSF - Macrophage colony-stimulating factor, CCL2 - Monocyte chemoattractant protein 1 (MCP1), IL1 – Interleukin1, IL3 – Interleukin 3, IL4 – Interleukin 4, IL6 – Interleukin 6, IL10 – Interleukin 10, MCP5 - Monocyte chemotactic protein 5 (CCL12), MIFα- Macrophage Migration Inhibitory Factor α, ROS - , VEGF - Vascular endothelial growth factor α, INFy – Interferon γ, PGE2 - Prostaglandin E, IDO - indoleamine 2,3-dioxygenase, TNFa - tumor necrosis factor α, S100A9 - S100 calcium-binding protein A9.

It is currently unclear whether tumor resection can affect tumor-associated phenotype of monocytes. The decrease in cytokine production observed during surgery may occur due to the effects of anaesthetics, and the increase in cytokine production after surgery may reflect surgical stress (82, 83). Surgical intervention leads to an increase in the level of IL-6 and IL-1 in peripheral blood after surgery (**Figure 1A**). TNF-α production is detected only during surgical interventions, that is associated with intestinal ischemia (82). In meta-analysis of 15 studies of breast cancer patients which compared methods of general anaesthesia and general anaesthesia combined with continuous paravertebral block, the level of CCL2, TNF-a and IL-8 was notably decreased in case of combined method (84). It can be assumed, that there is an effect of these cytokines on monocyte precursor cells in the bone marrow. However, the direct effect of surgical treatment on precursor cells has not been studied, and major focus was on the changes in the content of monocytes in blood.

#### Effect of surgical treatment on circulating monocytes

The number of peripheral blood leukocytes is increased significantly, and the number of lymphocytes is decreased in patients with stage T3 of colon cancer after surgery, indicating that surgical trauma and stress trigger systemic inflammation and suppress immune defense mechanisms (85). It is known that monocytic MDCS are characterized by low HLA-DR expression, and are usually considered as immunosuppressive cells (86). In patients with prostate and colorectal cancer, the percentage of immunosuppressive CD14+HLA-DR^low^ monocytes was elevated compared to normal individuals (80). Postsurgical normalization of CD14+HLA-DR^low^ cells occurred in prostate cancer patients, but not in patients with colorectal cancer (87). In patients with both cancer types, the interferon-γ response of T lymphocytes to phorbolmyristate acetate-ionomycin was increased compared with normal donors, but it was further increased after tumor ablation only in prostate cancer patients (87). It remains to be questionable whether CD14+HLA-DR^low^ cells were able to suppress inflammatory reactions, or their elevated levels were rather an attempt of the organism to compensate the disbalanced immune status in cancer patients. After glioblastoma resection, HLA-DR expression on monocytes was dynamically decreasing during first days after surgery, and returned to pre-surgery level after day 8 (88). In patients with lung cancer, thoractomy induced an increase in monocytic MDCS with CD11b+CD14+CD33+HLA-DR- phenotype in the blood compared to the status before surgery, and their accumulation correlated linearly with an increase in Treg amounts in circulation (89). Moreover, these surgery-induced MDCSs were more potent in expending Treg when co-cultured with autologous T cells *in vitro* (89). Mean number of monocytes along with their phagocytic activity were increased after surgery reaching the control point before treatment in breast cancer patients (90). In colorectal cancer patients, surgical intervention led to the reversal expression of previously upregulated genes (*ACP5, ADM, APP, BAX, CD68, CTSZ, CXCR4, FCER1A, FKBP5, GPER, HBA1, HBB, HLA-DQ1, HMOX1, HP, ILR2S100P, SLPI,SOCS3, TKT*) to the levels comparable to healthy individuals (15). In gastric cancer patients, post-surgery increase in monocyte count was a marker for early relapse (91). However, in oesophageal squamous cell carcinoma and lung adenocarcinoma post-surgery monocyte count positively correlated with overall survival and disease-free survival (92, 93).

#### Surgery affects the migration and functions of monocyte-derived cells

Postoperative monocytes migration is potentially important factor of recurrence-free period in cancer patients. Since tumor is eliminated by surgery, recruitment of monocytes into the site of surgery is not in focus of the investigators. However, it is reasonable to take under consideration the dynamics of circulating levels of major chemotactic factor for monocytes, CCL2 (**Figure 1A**). Thus, in patients with gastric cancer after palliative surgery, serum CCL2 levels decreased postoperatively, whereas in patients who underwent curative surgery, serum CCL2 level tended to increase (94). Surgery was shown to increase MDSC in circulation and in tumor tissues in animal tumor models and in patients with lung cancer, breast cancer, and melanoma (89, 95). Levels of major immunosuppressive mediators, indoleamine 2,3-dioxygenase (IDO) and arginase, was increased rapidly in circulating CD14+CD33+HLA-DR− cells after gastrectomy in gastric cancer (**Figure 1A**) (91). *Ex vivo*, isolated out of blood CD14+CD33+HLA-DR− cells suppressed INFγ production by CD8 T-cells (91). Gastric cancer patients with septic complications on 8th and 14th days after esophagectomy had alterations in monocyte phenotype and functions, such as increased H_2_O_2_ production and decreased HLA-DR expression *ex vivo* (85). After colon tumor resection, monocytes in *ex vivo* conditions had supressed ability to produce pro-inflammatory cytokines and chemokines directly after surgery (96). After three days *ex vivo*, reduced amount of IL1b was produced in response to LPS by patients’ monocytes. In addition, the authors detected increased expression levels of suppressors of cytokine signalling (SOCS)1 and SOCS3 mRNA (96).

Overall, in several types of solid tumors tolerogenic status of immune system, that is a natural way to resolve efficiently surgical trauma -induced inflammation, can affect both phenotypes of circulating monocytes or monocyte-derived MDSCs, as well as their ability to modulate inflammation and give signals to T-cells.

### Radiotherapy

Radiation therapy (RT) is considered as a standard in the treatment of most of solid tumors (97). Radioresistance is a key factor for RT failure, and development of locoregional relapse and distant metastases are the major obstacles for the success of the treatment (98–101). The involvement of TME in radioresistance was demonstrated in several studies (102, 103). One of the first studies on the RT effect on monocyte differentiation was performed by E. S. Buescher and J. I.Gallin in 1984 (104). They found that radiation doses used to prevent graft-versus-host reactions during leukocyte transfusions have detrimental impact on the survival and function of human monocytes *in vitro* (104). The long-term effects of radiation on monocytopoiesis are characterized by prolonged increase in the number of circulating monocytes, as it was demonstrated in Hiroshima and Nagasaki citizens in 50 years observation (105). It results in severe hematopoietic dysfunction, concomitant with a reduction in phagocytosis and deficiency in antigen recognition by T cells, antibody production and impaired activation of neutrophils and monocytes (106, 107). Localized irradiation affects healthy tissue that are adjacent to the irradiation site, with about half of the localized dose being absorbed by healthy tissues and bones (108). Local tissue destruction has more pronounced extend compared to the systemic one. After irradiation, the production of cytokines IL-1α/β, IL-6, IL-17, TNF-α, and VEGF is increased, and cellular senescence in a blood is levated (**Figure 1B**) (109–111). Below we present accumulated data about the effect of irradiation on monocyte biology in peripheral blood and tissues.

#### Radiotherapy affects progenitor cells

The effect of ionizing radiation depends on the total dose and fractionation rates (112). The direct action of radiation is related to the transfer of high energy to cells, resulting to membrane destruction and DNA damage followed by functional cell deficiency (113). Progenitor cells have high proliferative ability, and they are highly susceptible to irradiation. Overall, it was found that murine monocyte progenitors exhibit the impaired DNA repair activity and higher sensitivity to RT compared to macrophages. Total body irradiation in mice induced pronounced apoptosis in bone marrow-derived monocytes, but not in peritoneal macrophages (114).

Ionizing radiation promotes the suppression of immunity and subsequent bone marrow depression (**Figure 1B**) (115). Within first 24 hours after irradiation, active apoptotic death of hematopoietic cells results in monocytopenia (116). This effect is associated with a high sensitivity of monocyte progenitor cells to even low doses of irradiation (107). After irradiation at 10 Gy, only 1-3 out of 1000 stem cells, which are able for the regeneration, remain intact (117). Other stem cells in bone marrow are destroyed, and autologous recovery takes long time (117–119).

Irradiation toxicity can lead to spleen damage and decrease in the number of monocytes and macrophages contributing to tumor progression *via* reducing immune surveillance and pro-inflammatory stimulation (120, 121). Irradiation reduces not only spleen cellularity, but also the ability of survived lymphocytes and monocytes/macrophages to respond to mitogens (122). In spleen, the level of pSTAT1 and pSTAT4, which are activated by IFN-γ and IL-12, respectively, was significantly reduced by gamma irradiation, while an activation of STAT6 mediated by IL-4 receptor remained unchanged (**Figure 1B**) (123).

#### The effect of radiotherapy on the viability and recruitment of circulating monocytes

Analysis of RT effects on lymphoid and myeloid linage cells in healthy donors and patients with acute leukemia, who received allogeneic stem cell transplantation, revealed that monocytes are more resistant to RT than lymphocytes and granulocytes (106). The same tendency was found for patients with medulloblastoma (124). Irradiation negatively affects monocytes, significantly reducing their survival, which depends on the radiation doses. Thus, 2 Gy does not have significant impact on the number of circulating monocytes and monocyte-derived macrophages (125–127). In hybrid model of cross between female BALB/c and male DBA/2 CD2F1 mice, decrease in the percentage of circulating monocytes was detected four hours after irradiation at 8 Gy. After one month, monocytes were recovered and reached the pre-treatment level (128). Similar results were obtained in other studies. *In vivo* the number of circulating monocytes was noticeably decreased on the first days and was restored on days 21-28 after radiation (128–130) (**Figure 1B**). In patients with lung, breast, cervix and prostate cancers, who underwent RT (35 Gy), the number of circulating monocytes and eosinophils was significantly increased, that can be explained by the compensatory mechanisms activated by irradiation (131). In patients with larynx cancer, a decrease in the lymphocytes/monocytes index was observed from the point before RT to 6 weeks after RT (67). In patients with Hodgkin’s lymphoma, lymphocytes, neutrophils and monocytes are severely decreased immediately after irradiation at 35 Gy. Monocytes recovered quickly to ~ 50% pre-therapy level, and increased transiently to pre-therapy level up to 3 months after RT (132). Patients with medulloblastoma, who received a total of 35 Gy, had wave-like decrease and recovery of monocyte count that reached pre-treatment level to the end of treatment, maintaining this plateau for 2 months. In independent cohort, using a lower dose of 12.5 Gy leaded to increased amount of monocytes compared to pre-treatment level (132). In breast cancer patients, monocyte count was significantly higher 3 months after radiotherapy, compared to the pre-treatment level (133). These data indicate that depletion of monocytes is compensated during therapy.

As was mentioned above, peripheral blood monocytes are hypersensitive to IR and display high rate of apoptosis even after exposure at low doses (<2 Gy), compared to monocyte-derived macrophages which are hyposensitive. Monocytes have higher levels of single-chain and double-strain breaks and lower rate of DNA repair compared to macrophages (134). It can be explained by instability of monocytes to reactive oxygen species (ROS) impact, resulting in the protein oxidation and cell death independently of caspase-3 that induces monocyte necrosis (135). Monocytes and macrophages differentially respond to ROS-mediated oxidative stress. The sensitivity of monocytes to ROS is associated with phosphorylation of genes involved in DNA reparation, such as *ATM, ATR, CHK1, CHK2* and *p53*, and activation of caspases 3, 7 and 8, which were not observed in macrophages and dendritic cells (**Figure 1**) (134).

Ionizing radiation can change migratory ability of monocytes *via* systemic stimulation of chemoattractant factors (136–140). Cytokine analysis in murine models for prostate and pancreatic cancer revealed elevated levels of M-CSF and CCL2 in the irradiated tumors and in circulation that stimulated recruitment of bone marrow and spleen-derived monocytes into tumor tissue (**Figure 1B**) (136, 141). Low-dose radiation increased serum level of anti-inflammatory cytokines IL-3 and IL-4, and pro-angiogenic vascular endothelial growth factor (VEGF) needed for efficient tumor vascularization (138). Circulating levels of chemotactic factors for monocytes and macrophages CCL2, MCP-5 and MIP-α were also elevated. At the same time, the levels of pro-inflammatory cytokines IL-12p70, IL-13, IL-17 and IFN-γ were decreased in mice circulation (138). This profile of circulating factors suggests that despite retaining high potential to be recruited into tumor mass, monocytes can be programmed in circulation to tolerate the tumor. Enhanced macrophage migration that was induced by conditioned medium from irradiated prostate cancer cells was completely blocked by a selective CSF1R inhibitor (136). In patients with prostate cancer, the level of CSF1 in blood serum was increased after RT (**Figure 1**) (136). The selective CSF1R inhibitor combined with RT suppressed tumor growth *in vivo* more effectively than RT alone (136). In murine models of head and neck tumor, breast cancer, and pancreatic ductal adenocarcinoma, response to RT was accompanied by CCL2 production, resulting in the recruitment of Ly6C^+^CCR2^+^ monocytes to support tumor proliferation and neovascularization after RT (139–141). The disruption of CCL2-CCR2 axis by *ccl2* knock-out or by using CCL2 neutralizing antibody in combination with RT increased the efficiency of RT in head and neck, breast, and pancreatic ductal adenocarcinoma (**Figure 1B**) (139–141). Besides well-known CCL2/CCR2-mediated mechanism, monocyte infiltration into tumors after ionizing radiation depends on nerve damage-induced protein 1 gene (Ninjurin1, or Ninj1) which encodes a hemophilic adhesion molecule, and is activated in inflammatory lesions (142). Tumor hypoxia created by radiation-promoted vasculature disruption and enhanced activities of HIF-1/HIF-2 induced various cytokines/chemokines expression by residual tumor cells and tumor stromal (143). Also, hypoxia-induced HMGB1 facilitates the increase in the amounts of immunosuppressive cells such as MDSCs, and promotes the re-polarization of M1-type macrophages to M2-type macrophages (144). As a result, hypoxia enhances accumulation of immune cells and creates optimal conditions for the differentiation tumor-supporting monocyte-derived cells.

#### Radiotherapy affects monocyte differentiation and function

As described above, sensitivity of monocytes to irradiation is decreased during their differentiation into macrophages. This is associated with the ability of progenitor cells to actively proliferate compared to more differentiated cells (106). Human macrophages obtained from THP-1 monocytic cells are highly resistant to radiation-induced apoptosis compared to undifferentiated THP-1 cells *in vitro* (145). The phagocytic activity of human monocytes and macrophages *in vitro* was inhibited by irradiation at doses of 2500 and 5000 rad (104). After irradiation of 55 and 650 rad, phagocytic ability of human monocytes isolated from peripheral blood was not significantly affected by ionizing radiation, and monocytes were more radioresistant than lymphocytes (146). Phagocytic activity of peripheral blood monocytes remained intact in breast cancer patients undergoing RT at 5400-6640 Gy (147).

The influence of irradiation on immune cell activation can reflect hormesis phenomenon provided beneficial effects on organism (148). RT effect on the tumor-associated phenotype of macrophages was demonstrated in several studies, but the mechanism of this effect remains unclear. For example, increase in the expression of pro-inflammatory markers *CD86, HLA-DR* and *CD80*, but not *CCR7, TNF and IL1B*, and inhibition of the expression of anti-inflammatory markers *CD163, MRC1, VCAN* and *IL10* were observed after 10 Gy exposure of human monocyte-derived macrophages, indicating a modulation of cell phenotype towards more pro-inflammatory one (149). These irradiated macrophages had increased phagocytic activity, and contributed to the invasion of cancer cells and angiogenesis *in vitro* (149). In THP-1 and RAW264.7 cell lines, radiation induced the expression of pro-inflammatory interferon-regulated factor 5 (IRF5) and an activation of a mutant kinase of telangiectasia ataxia (ATM), which are also crucial for the pro-inflammatory polarization of macrophages (150). Irradiation of human monocytic cell line U937 by γ-rays using a 137Cs source with a fixed dose rate of 148 or 531 cGy/min promoted antigen-presenting functional maturation by phosphorylating NFκB family member (151). In human monocytes isolated from healthy volunteers, low doses of irradiation (0, 05 and 0,1 Gy) activated MAPK pathways and positively regulated TLR signaling (152). Inhibition of NOS2 and ROS as well as an increase in arginase activity in bone-marrow macrophages were detected after the irradiation at dose 0.45 Gy/min in non-tumor immunodeficient C57BL/6 mice (153). In C57Bl/6J murine model of prostate cancer, irradiation induced upregulation of *Arg-1* and *COX-2* within 5 days and activation of iNOS-arginine pathway after 10 fractions of 25Gy irradiation in cells of TME, particularly in TAMs (**Figure 1**) (154). Expression of HLA-DR was up-regulated in monocytic DCs during hypofractionated RT treatment in colon cancer patients (155). Moreover, RT had two opposite effects on MDSCs, depending on the dose, fractionation scheme, and tumor model. Fractionated radiation therapy increased MDSCs amounts in tumor tissues, while ablative hypofractionated radiation therapy decreased MDSCs (156, 157). However, regardless of the stereotactic body RT regimen, several studies have shown that targeting MDSCs by Sunitinib, a multitargeted tyrosine kinase inhibitor of VEGFR1, VEGFR2, VEGFR3, PDGFR, c-kit, FLT3, and RET, increases the anti-tumor effect of RT (158).

Thus, radiation therapy impairs hematopoietic pool of progenitor cells due to their high proliferative activity. The damaging effect of RT declines from circulation monocytes to the differentiated monocyte-derived macrophages. Systemically, RT alters monocyte functions: proliferation, growth and phagocytic activity. Monocyte-derived macrophages, DCs and MDSCs are significantly more resistant to RT and respond to irradiation mainly by switching their phenotype toward tumor-supporting one.

### Chemotherapy

Chemotherapy (CT) is one of the main anti-tumor treatment strategies, based on the cytotoxic effect of chemical drugs with different mechanisms of action on tumor cells suppressing their proliferation and inducing apoptosis (71). Systemic CT can affect hematopoietic progenitor cells, circulating immune cells and cells recruited from the bloodstream to tissues (1, 73). Collected data demonstrate that CT has an impact on the amount of circulating monocytes and tumor cell-TAM interactions. Understanding the dynamics of monocyte populations during CT can be helpful for the search of significant markers of therapy response and molecular targets to improve CT efficacy.

#### Chemotherapy modulates monocyte entry in the blood circulation and recruitment to tumor sites

Monocyte recruitment both in peripheral blood and tumor tissue is multifunctional process related to chemotactic activity of tumor cells and components of TME, that can be modulated by chemotherapeutic impact (2, 19, 159). CT acts systemically, and cytostatic effect is directed to tumor cells and hematopoietic cells simultaneously. Progenitor cells have a greater ability to proliferate and are more sensitive to CT (1, 160).

Elevated TAM infiltration after taxane-based chemotherapy was demonstrated in patients with breast cancer and glioblastoma (161, 162). Monocyte recruitment to tumor site was increased in MMTV-PyMT (mouse model of breast cancer metastasis) treated with doxorubicin (163). Taxanes and anthracyclines promoted the secretion of tumor-derived extracellular vesicles, enriched with annexin A6 (ANXA6), stimulating CCL2 production and Ly6C+CCR2+ monocyte expansion in the pulmonary pre-metastatic niche in a breast cancer model (**Figure 1C**). Monocyte accumulation in turn facilitated the development of lung metastasis (164). The amount of monocyte progenitors in bone marrow and in spleen of Balb/c mice significantly decreased after 2 days of cyclophosphamide treatment. By day 7, the total amount of cells in bone marrow and spleen returned to the pre-treatment level (165). M-CSF (or CSF1) is a crucial factor of monocyte progenitors’ differentiation (166). Paclitaxel increased the expression of CSF1 and IL-34 in tumor cells, followed by the recruitment of large number of CSF1R-expressing monocytes in mouse mammary tumors (**Figure 1C**) (161). Treatment with paclitaxel and carboplatin increased the expression of CCL2, a key chemokine for monocyte recruitment, in ovarian cancer cells activating monocyte migration *in vitro* and *in vivo* (167). The combination of chemotherapy, irradiation and vascular destroying agents (such as combretastatin-A4-P) resulted in increased production of CSF1 and CCL2 along with CXCL12 by tumor cells, that induced monocyte recruitment in Lewis lung carcinoma (LCC) model and MMTV-PyMT model (168). In breast cancer patient-derived xenograft model, carboplatin and taxane-based CT stimulated monocytosis followed by systemic elevation of CCL2 level (169). Treatment with doxorubicin, docetaxel and SP600125, an c-Jun N-terminal kinases (JNK) inhibitor, stimulated CCL2-depended monocytosis that supported breast cancer stem cells proliferation (170). ShRNA and pharmacological suppression of the CCL2-CCR2 pathway diminished chemotherapeutic adverse effect on cancer stem cells (**Figure 1C**) (169, 170). YLK39, a novel strong chemotactic factor for primary human monocytes, whose expression in tumors after neoadjuvant chemotherapy predicted an increased risk of distant metastasis and poor response to preoperative CT (170).

Since monocytes replenish the pool of TAMs with tumor-supporting activity, blocking monocyte recruitment can be potentially used as a therapeutic strategy. Inhibition of monocyte renewal and recruitment by anti-chemotactic molecules improved the response to CT in numerous studies. Anti-CSF1R antibody depleted TAMs in mouse models of colorectal adenocarcinoma, fibrosarcoma, breast cancer, hepatocellular carcinoma, pancreatic cancer and glioma (171–175). Anti-CSF1R treatment enhanced response to clinically used chemotherapeutic agents such as paclitaxel and gemcitabine in mammary and pancreatic ductal adenocarcinoma murine tumors (161, 176). Similarly, the combination treatment with anti-CCL2 (carlumab, CNTO 888) and conventional chemotherapy (docetaxel, oxaliplatin and irinotecan plus folinic acid and 5-fluorouracil [5-FU]) resulted in the increase of the antitumor response in prostate and pancreatic cancers (177). Most of studies are limited to the analysis of CCL2/CCR2 interaction which was a target for a number of clinical trials. However, these clinical trials have never reached phase III level in cancer according to clinical trial data on ClinicalTrials.gov. Failure of clinical trials can be explained by number of redundantly acting chemokines attracting monocytes into pathologically change tissue, the mechanism that evolutionary needs high level of safety due to the key role in host response against pathogens.

Thus, CT induces production of factors that stimulate monocyte progenitors’ proliferation and monocyte entry into the circulation*. Since newborn monocytes are chemotherapy-intact, their repolarization after CT course can be promising approach for anti-tumor therapy in adjuvant regimen. This point needs to be investigated more thoroughly.

#### Chemotherapy affects monocytes in peripheral blood

The dynamics of the count and subset composition of monocytes during CT treatment has been studying for several years due to easy access to monocytes in peripheral blood (178–180). Normal monocyte count is 400/µl (0.4 × 10^9^/liter) for healthy adults. Men tend to have slightly higher monocyte counts compared to women. Increase in the number of blood monocytes correlates directly to the raise in a total pool of monocytes and a monocyte turnover rate. The pool of peripheral blood monocytes is renewed every 5 days (181).

Chemotherapy seems to have less effect on mature monocytes. The count of these monocytes can decrease due to a lifespan termination in peripheral blood leading to complete exhaustion called the nadir. Renewed monocyte population after CT treatment may have a different functional profile with the loss of pro-tumor programming in case of favorable outcome.

In cancer patients, nadir point for monocytes is observed at 5-7 day after CT drug injection (178–180) (**Table 2**). In patients with pancreatic cancer, the number of CD14+ cells has been significantly decreased at day 14 after gemcitabine treatment, and then recovered until day 55th (178) (**Table 2**). In BALB/c mice, that received 4 doses (on day 1, 7, 14 and 21) of doxorubicin or docetaxel, monocyte count was decreased from day 6 to day 21, and then raised up until day 45 (185). Absolute number of white blood cells was decreased, but monocyte absolute count had no significant changes during five cycles of doxorubicin plus cyclophosphamide treatment in luminal B and Her2+ breast cancer patients. After five CT cycles, monocyte absolute count was drastically decreased, concomitant to the switch to paclitaxel treatment (183) (**Table 2**). In the same patients, decrease in absolute neutrophil and leukocyte counts as well as leukocytes-monocytes ratio was observed from 1st to 5th cycle of CT treatment with significant increase to the end of 8th cycle (183). Due to having a common progenitor, monocyte and neutrophil counts correlated during CT (186). Wherein monocytopenia preceded neutropenia by 4-6 days, that was a predictive factor for side effects after docetaxel, cisplatin and fluorouracil-based therapy in patients with head and neck cancer and with lung cancer (186). Considering that the vulnerability to infectious and febrile neutropenia are frequently the limiting adverse effects of anti-cancer CT and can cause life-threatening events, they can be useful indicators for modulating treatment regimens (187).

**Table 2:** Dynamics of circulating monocytes during chemotherapy in cancer patients.

Cancer type	Number of patients	Parameter	Chemotherapy	Dynamic	Reference
Lung, Breast, Esophagus, Nasopharynx cancers	103	absolute monocyte count	Docetaxel+Cispaltin Gemcitabine+Cisplatin Vinorelbine+Cisplatin Measurements after single CT dose and G-CSF* treatment after 10 days	Decrease during first 4-6 days (FCh - 3.81, p-value < 0.001 ) Nadir at 5,65^th^ day Increase until day 24 (FCh – 2.36, p-value < 0.001 compared to nadir day )	(180)
Lung cancer	75	absolute monocyte count	Carboplatin or cisplatin Measurements after single CT dose	Decrease during first 5-7 days (FCh - 9) Nadir at 6^th^ day Increase until day 15 (FCh – 9compared to nadir day)	(182)
Pancreatic cancer	18	absolute monocyte count	Gemcitabine Days of gemcitabine treatment: 1^st^-7^th^ - 14^th^ 28^th^ –35^th^-42^th^	Decrease during first 5-7 days (FCh – 3.5, p-value< 0.001 ) Nadir at 7^th^ day Increase until day 30 (FCh – 4, p-value< 0.005 compared to nadir day) Decrease until 36^th^ days (FCh – 4, p-value< 0.001 compared to 30^th^ day ) Nadir at 36^th^ day Increase until day 55 (FCh – 2, p-value< 0.05 compared to 36^th^ day)	(178)
		CD14+ monocytes		Decrease during first 15 days (FC – 2.5, p-value< 0.01 ) Nadir at 15^th^ day Increase until day 28 (FCh – 3 compared to nadir day) Decrease until day 55 (FCh – 1.5, compared to 28 day and FCh – 1.5, p-value< 0.01 compare to before treatment)	
Breast cancer	20	absolute monocyte count	Doxorubicin + Cyclophosphamide (4 cycles) Days of treatment: 1^st^-21^st^ – 42^d^-63^d^ Paclitaxel (4 cycles) 84^th^-105^th^-126^th^-147^th^	Increase from 1^st^ to 5^th^ cycles (FCh – 1.5) Dramatically decrease after 5^th^ cycle (FCh – 6) Increase until 8^th^ cycle (FCh – 1.2)	(183)
		CD14+16- monocytes		Increase from 1st to 3th cycles (FCh – 1.5, p-value< 0.05) Decrease from 3^th^ to 5^th^ cycle (FCh – 1.2, p-value< 0.05) Dramatically decrease after 5^th^ cycle (FCh – 2.5, p-value< 0.05) Increase until 8^th^ cycle (FCh – 2, p-value< 0.05)	
		CD14-16+ monocytes		Decrease from 1^st^ to 4^th^ cycles (FCh – 1.5, p-value< 0.05) Increase from 4^th^ to 5^th^ cycle (FCh – 1.2, p-value< 0.05) Dramatically increase after 5^th^ cycle (FCh – 2, p-value< 0.05) Decrease until 8^th^ cycle (FCh – 1.5, p-value< 0.05)	
Triple-negative breast cancer	56	CD14+16- monocytes	Nanoparticle albumin-bound paclitaxel (Nab-Pac) and by epirubicin and cyclophosphamide Days of treatment: Every week (x12) of Nab-Pac Every 2 weeks (x4) epirubicin and cyclophosphamide	Decrease until 12^th^ cycles (FCh – 1.2, p-value ≥ 0.05) Increase until last 4^th^ cycles (FCh – 1.7, p-value< 0.05 compare to12^th^ cycle)	(184)
		CD14+16+ monocytes		Decrease until 12th cycles (FCh – 1.2, p-value ≥ 0.05) Increase until last 4th cycles (FCh – 2 p-value< 0.05 compare to12th cycle)	
		CD14-16+ monocytes		Decrease until 12^th^ cycles (FCh – 1.7, p-value ≥ 0.05) Increase until last 4^th^ cycles (FCh – 2.4, p-value < 0.05 compare to12^th^ cycle)	

G-CSF, granulocyte colony-stimulating factor. FCh; Fold change.

Chemotherapeutic agents can affect monocyte subpopulations in circulation blood (160, 188, 189). In breast cancer cohort, the amount of CD3-CD14+CD16- monocytes was increased during 5 cycles of doxorubicin, dramatically decreased after 5^th^ cycle, but then has reсovered after following 3 cycles of paclitaxel treatment (183). For another breast cancer cohort, gradual decrease in CD14+ and CD14+HLA-DR^low^ subsets during 6 cycles of 5-FU, epirubicin, cyclophosphamide or capecitabine treatment was found (190). Non-classical subset (CD3-CD14+CD16^High^) of monocytes demonstrated the opposite trend in this study (190). In HER2+ breast cancer patients, a positive correlation between the amount of classical and non-classical monocytes and plasma IL-10 level was found during neoadjuvant chemotherapy (**Figure 1C**) (183). In patients with triple-negative breast cancer, significant decreased in classical, non-classical and intermediate monocytes was found after 12 weeks of treatment with nanoparticle albumin-bound paclitaxel. The monocyte count for all subsets increased after two weeks of treatment with epirubicin and cyclophosphamide, with maximum at week 8 (**Table 2**) (184). 5FU-based CT treatment of liver metastases in colorectal cancer patients induced rapid decrease in CD14+CD16+ intermediate subset of peripheral blood monocytes that predicted poor response to NAC (41). Etoposide plus cisplatin combination treatment elevated the amount of IL-10+CD206+CD14+ M2-like monocytes in peripheral blood of patients with small cell lung cancer (SCLC) compared to non-treated patients (191). In non-small cell lung cancer (NSCLC) patients, both frequency and absolute number of CD14+HLA-DR^-/low^ cells were significantly increased in the peripheral blood compared to healthy control. Increased amount of CD14+HLA-DR^-/low^ monocytes was indicative for poor response to platinum-based chemotherapy and short period of PFS (192). In advanced-stage ovarian cancer patients, CD14+HLA‐DR^low/−^ monocytes were diminished by platinum and taxane-based chemotherapy (193).

In addition to the repopulation of monocytes in peripheral blood, their proprieties can also be changed during CT treatment. In cyclophosphamide-treated mice, monocytes possessed gene expression signature specific for neutrophil precursors, defined by increased proliferative capability and elevated amounts of multiple granules and suppressed T-cell proliferation *in vitro* (165). Two days after CTX there was a sharp decrease in cellularity in both bone marrow and spleen, and the total number of cells returned to a baseline level by day 7 (165). In mice, HIF-1α–deficient tumors had reduced numbers of proangiogenic TEMs, and tumor were also significantly smaller after docetaxel chemotherapy (194).

The prognostic and predictive values have been demonstrated for lymphocyte to monocyte ratio (LMR). Increased pre-treatment absolute lymphocyte count and high LMR were independent favourable prognostic factors in patients with metastatic nasopharyngeal carcinoma (67). Positive correlation between pre-treatment LMR and pCR was observed in patients with locally advanced oesophageal squamous cell carcinoma who underwent neoadjuvant platinum-based chemoradiotherapy (195). Significant correlation between lower monocyte count after NAC and pCR was found in triple-negative breast cancer patients (196). Increased IL-10 level and monocyte counts correlated with lower rates of pCR (183). High percentage of CD11b+CD14+ and CD11b+CD14+S100A9+ cells predicted poor response to platinum-based chemotherapy and negatively correlated with PFS in patients with NSCLC (197). Oppositely, in triple negative breast cancer patients who received non-platinum-containing treatment, monocyte counts positively correlated with disease-free survival (196).

Thus, similar kinetics of increase in monocyte amounts in circulation was observed in cancer patients treated by various chemotherapeutic drugs. The nadir point for monocytes precedes the one for neutrophils. After CT application, the number of circulation monocytes can come down to the original level within several week. It can be assumed that newly differentiated monocytes carry genetic, epigenetic and transcriptional modifications developed under the influence of cytostatic drugs. Most advanced single cell resolution technology provides now a useful tool to decipher the impact of each programming level on functionality of monocytes when they enter tumor mass.

#### Chemotherapy affects monocyte differentiation

The most important stage of the ontogenesis of monocyte-derived TAMs, DCs and MDCSs which can be modulated by CT treatment is tumor-supporting differentiation (11, 198, 199). In MDA-MB435 breast carcinoma mouse model, paclitaxel+albumin treatment resulted in increased infiltration of tumor by CD45+CD169+ and F4/80+ macrophages (200). The expression of GM-CSF, a crucial growth factor for monocyte to macrophage differentiation, was significantly upregulated in various pancreatic ductal adenocarcinoma cell lines or human pancreatic ductal adenocarcinoma tissues after gemcitabine treatment. In patients with pancreatic ductal adenocarcinoma, GM-CSF expression correlated to poor prognosis after CT treatment (**Figure 1**) (201). The blockade of GM-CSF with monoclonal antibodies facilitated T-cell proliferation in co-culture of monocytes stimulated by tumor cell-conditioned supernatants and T lymphocytes (202). Most chemotherapeutic agents used in conventional cancer chemotherapy reduce the number of MDSCs in tumor tissues and peripheral lymphoid organs, and inhibit differentiation in MDSCs (203). Gemcitabine treatment stimulated the immunosuppressive activity of monocytic MDSC and, accordingly, decreased the amount of IFN-γ-producing CD4+ and CD8+ T cells in mammary tumor-bearing mice (204). Cyclophosphamide treatment induced the expansion of inflammatory monocytic myeloid cells *in vitro* that attenuated antitumor CD4+ T-cell response through the PD-1-PD-L1 axis (165). Treatment with cisplatin or carboplatin increased the production of IL-6 and PGE2 by cervical cancer cells, head and neck cancer cells, and myeloma cell lines thus promoting STAT3-dependent IL-10 production by M2 macrophages with tumor-supporting activity (**Figure 1C**) (205). In patients with breast cancer, high expression of monocyte chemotactic factor YKL-39, specifically expressed by TAMs, correlated with increased risk of distant metastasis and poor response to PTX- or taxotere-based NAC (170). In breast cancer patients, CT did not affect monocyte amounts, but phagocytic index of monocyte-derived macrophages was diminished (90). In breast cancer patients who underwent anthracycline-containing NAC, the absence of clinical response was associated with the presence of M2-like macrophages with YKL-39-CCL18+ or YKL-39+CCL18- phenotypes (206). These data indicate that CT aggravates immunosuppression by differentiation of monocytes toward tumor-promoting macrophages.

Thus, cytostatic agents are able to modulate the recruitment of monocytes into the tumor, their differentiation into specific TAM populations, and their regulation of adaptive antitumor immune responses. Such mode of action can dramatically affect tumor progression after chemotherapy, contributing to poor response and poor outcome. In future, combined chemotherapeutic and immunomodulatory approaches based on the modulation of monocyte-TAM activity can help to increase CT efficacy.

### Immunotherapy

Immunotherapy is one of the promising targeted strategies for the treatment of malignant neoplasms (207–211). Immunotherapy can be divided into two main types - active or passive. Active immunotherapy uses CAR-T cells, cancer vaccines and monoclonal antibodies (mAbs) that specifically target tumor cells through the immune system. Passive immunotherapy does not directly affect tumor cells, but increases the ability of the immune system to attack them. The latter type includes checkpoint inhibitors and cytokines. Patients treated with immunotherapy showed different response rates depending on cancer type and cohort composition with the same malignancy (212, 213). The impaired anti-tumor immune response observed in patients with decreased immunotherapy efficacy is likely a consequence of immune system dysfunction: diminished antigen presentation/detection, leukopenia, and coordinated network of immunosuppressive cell surface proteins, cytokines and cellular mediators (214). The use of autologous monocytes, and interferons alpha and gamma for local-regional administration directly into the peritoneal cavity demonstrated first promising results for improving immune response (215).

Immune checkpoints are inhibitory receptors and pathways that are responsible for maintaining self-tolerance and modulating immune response (216). One of the most used immune checkpoint inhibitors (ICI) include anti-PD-1 or anti-PD-L1 molecules, which prevent the blocking of the immune system and activate attacking of tumor cells by T lymphocytes. PD-1 is an inhibitory receptor expressed on activated T cells, B-cells and monocytes (217). Drugs in this category currently include nivolumab (Opdivo), atezolizumab (Tecentriq), pembrolizumab (Keytruda), avelumab (Bavencio), durvalumab (Imfinzi) and cemiplimab-rwlc (Libtayo). Despite that these drugs target T-cells, they are not fully effective in the absence of myeloid cells such as monocytes and macrophages (218, 219). There is only limited information about IT influence to monocytes progenitors today. The combination of oxaliplatin, thymidine-based antitumor nucleoside analogue trifluridine and thymidine phosphorylase inhibitor tipiracil, which suppresses bone marrow, induced immune cell death *in vivo*, providing a rationale way for the use of these drugs to eliminate immunosuppressive cells in patients with metastatic colorectal cancer (**Figure 1D**) (220). We describe bellow the available data for IT effect on circulating monocytes and their differentiation in the tumor tissues.

#### Relationship between circulating monocytes and the effect of immunotherapy

In patients with nasopharynx cancer, breast cancer and esophageal cancer, increased absolute monocyte count and/or CD14+16- monocyte percentage in a pool of peripheral blood mononuclear cells correlated with worse response to anti-PD-1/PD-L1 therapy and decreased PFS (221). In melanoma, a higher content of CD14+CD16−HLA-DR^high^ monocytes before anti-PD-1 therapy is a strong predictor for PFS and OS (**Figure 1D**) (222). The average number of monocytes remained unchanged after ICI-based therapy compared to pre-treatment amount (223). Another ICI therapy is directed at CTLA-4, a cytotoxic protein 4 associated with T lymphocytes. Ipilimumab blocks the activity of CTLA-4 facilitating the elimination of cancer cells by the immune system (224). The amount of classical monocytes (CD14+CD16−) that express high levels of HLA-DR before treatment was predictive for improved OS in neuroblastoma patients (225). The majority of CD14+HLA-DR^low/neg^ cells are classical monocytes, so the higher expression of HLA-DR may reflect lower levels of immunosuppressive monocytic MDSCs (226). High level of CD14+HLA-DR^low/neg^ monocytes in peripheral blood of melanoma patients can be indicative for poor response to immunotherapy with anti-CTLA4 agent (**Figure 1D**) (227–229). Patients with lung and gastric cancers who have high PD-L1 expression on monocytes rarely respond to anti-PD1 therapy and have poor PFS and OS (230, 231).

Successful outcome of mAb therapy can depend on the initial number of monocytes in peripheral blood (226). At a primary low level of monocytes, the myelosuppression or decrease in the number of monocytes in tumor tissue after treatment may occur (226). Monocytes provide an essential mechanism for the depletion of malignant B cells under successful anti-CD20 therapy (232). Therefore, there is an urgent need to monitor the number and function of monocytes in patients with lymphoma undergoing anti-CD20 and other mAb-based therapies (232).

#### The effect of immunotherapeutic drugs targeting monocyte recruitment

Some drugs designed to inhibit monocytes/macrophages migration into tumor are also under investigations (233, 234). However, clinical trials were not as successful compared to CTLA-4 and PD-1/PD-L1 targeting (233, 234). Anti-S100A9 (Tasquinimod) treatment reduced tumor growth partially by reducing the recruitment of Ly6C^high^ cells from circulation to tumor tissue in mammary and melanoma mouse model (233). Anti-CCL2 antibodies were also proposed as a targeted immunotherapy for cancer. The main idea of using the drug was to block the recruitment of monocytes into the tumor. Anti-CCL2 CNTO 888 (carlumab) has demonstrated antitumor activity and good tolerance in cancer patients, but clinical trials on carlumab are not completed (235). Combination of carlumab with conventional chemotherapy are under clinical trials (236). However, recent results showed that increased metastasis rate was reported following anti-CCL2 therapy (237). In breast cancer model, the discontinuation of anti-CCL2 therapy was associated with descendance of monocytes from the bone marrow, increased mobilization of tumor cells from the primary site, an increase in their infiltration into the metastatic loci and increased angiogenesis promoted by IL-6 and VEGF (**Figure 1D**) (237).

#### Immunotherapy and differentiation of monocytes in tumor tissues

Chemotherapy in combination with ICI therapy induces upregulation of PD-L1 on cancer cells, facilitates infiltration of CD8+ T cells and NK cells, increases maturation of antigen-presenting cells including DCs and TAMs, and promotes MDSC activation (217). Mononuclear cells isolated from peripheral blood of healthy donors decreased the proliferation of nivolumab-treated T cells *in vitro* (225). Increased interactions of therapeutic mAb with monocyte receptors can enhance antibody dependent cellular cytotoxicity *in vivo* (238). Although most anti-CD20 antibodies activated the complement system *in vitro*, B-cell depletion was completely effective in mice with genetic insufficiency of C3, C4, or C1q complement components (232). The fact that monocytes deplete B cells *via* FcyR-dependent pathway during anti-CD20 immunotherapy has important clinical implications for tumor treatment with anti-CD20 mAbs (Rituximab) and other antibody-based drugs (239). Anti-CD20 mAbs induce the production of pro-inflammatory chemokines by immune cells inside the tumor, in particular by infiltrating macrophages (240). Rituximab can also lead to the release of pro-inflammatory cytokines, including TNF-α and IL-6, which can further enhance the immune response (**Figure 1D**) (240). Monocytes and macrophages induce antibody-dependent cytotoxicity and phagocytosis of tumor cells in the presence of antitumor mAb IgG (241). In pancreatic ductal adenocarcinoma model, macrophages activated by anti-CD40 mAb quickly penetrated tumor, acquired tumor-like phenotype and contributed to the depletion of tumor stroma *via* blocking CD40-expressing macrophages (242). Treatment with antibodies against immune checkpoint inhibitors a-PD-1 + a-CTLA-4 resulted in a dynamic remodeling of intra-tumoral monocyte-derived macrophages (241, 243). CyTOF and scRNASeq analysis identified 5 and 8 subsets of monocytes/macrophages respectively with difference functional direction that correlated with tumor progression (243).

Bispecific antibodies are proteins that target specific tumor antigens (similar to mAb) and bind proteins on the surface of immune cells (66). This allows T cells to kill cancer cells (244). Blinatumomab (Blincyto) is an example of a bispecific antibody (anti-CD3—anti-CD19) that recruits T cells to B cells. The drug was approved for the treatment of recurrent and refractory forms of B-cell malignancies, such as B-cell acute lymphoblastic leukemia and non-Hodgkin’s lymphoma (245). Catumaxomab targets EpCAM-positive carcinomas (246). A complex mechanism of action is related to an effective destruction of cancer cells, but excessive inflammatory background can be developed as a result of the pro-inflammatory cytokine production by blood monocytes (246).

Thus, immunotherapy effects can be mediated by monocyte responses. The effect of immunotherapy on monocyte activity can have both direct and indirect mode of action. The first one related to monocyte-specific alterations, and the second one is based on monocyte interactions with other cell types, e.g., CD8 T-cells or DC. The development of novel immunotherapeutic approaches aimed at molecular targets in monocytes can be also a promising strategy for improvement therapeutic effect.

## Genotype alteration in monocytes response to anti-cancer therapy

**Figure 2:**
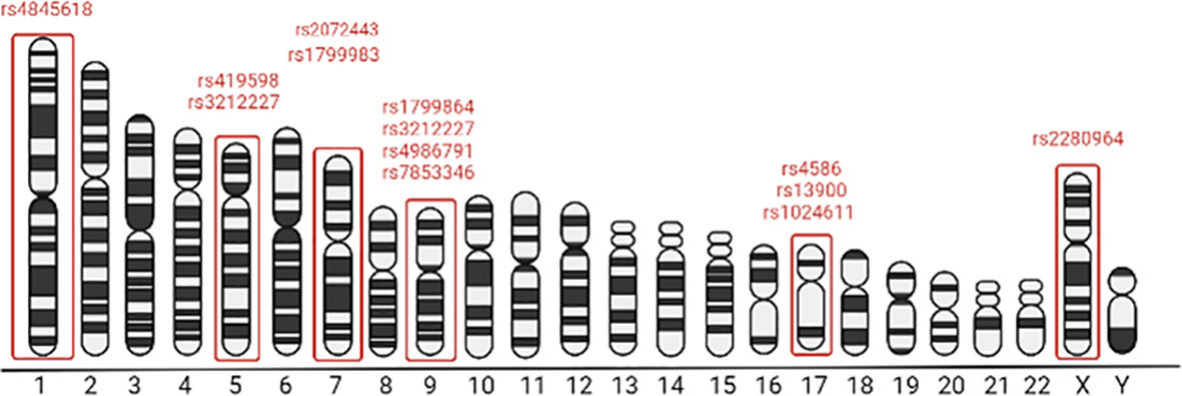
Chromosomal localisation of SNPs associated with the monocytes’ involvement in the cancer therapy effects. Figure created in biorender (http://biorender.io).

Constitutive features are one of the factors that can define the immune responses in general, as well as antitumor response of immune system. To date, there is no comprehensive view of the importance of gene polymorphic variants of myeloid cell in oncogenesis. It is known that the presence of single nucleotide polymorphisms (SNP) of the CCL2 gene is associated with breast, oral, endometrial, non-small cell lung, prostate and bladder cancers (56, 57, 60, 61, 247). Data on the correlation of SNP on monocytes with an influence of antitumor treatment are discrete and provide information about individual genes. We have summarized the monocyte-related SNP variants and their chromosomal localization on **Figure 2**. Thus, in monocytes, the Ala134Thr variant (rs2072443) in TMEM176B is associated with poor gene expression and with an increased activation of NLRP3 inflammasome. This SNP correlates with longer overall survival in colorectal cancer cohort (**Table 3**) (248). Patients with nasopharyngeal carcinoma with AA and AG gene variants showed a significant correlation with the development of distant metastasis after the initial RT (251). Moreover, rs4986790 and rs4986791 of *TLR4* significantly correlated with poor efficacy and early relapse in patients with breast cancer, that was confirmed using mice models and *in vitro* tests on human monocyte-derived DCs (**Table 3**) (250). SNP of *CCL2* is associated with longer PFS after conventional CT with anti-VEGF drug Bevacizumab compared to wild variants (**Table 3**) (249). Analysis of seven SNPs of *CCL2*, *NOS3, IL6R, IL12B, IL1RN* and *CXCR3* genes from patient cohort with different cancer types showed that SNP carriers have better response to anti-PD-1/PD-L1 treatment (**Table 3**) (253). Evidently, CCR2-depend pathway defines the response of brain tumor cells to radiotherapy (**Table 3**) (254). Thus, several studies about single polymorphic gene variants describe correlations between monocyte SNPs and CT and RT efficacy. Therefore, new technologies are required to clarify the issue of the influence of genetic features on the reactions of monocytes to anti-cancer therapy.

**Table 3:** Monocyte SNP association with anti-cancer therapy effects.

Therapy	Gene, SNP	Cancer type	Correlation	Reference
Surgery	*TMEM176B *rs2072443 (chr.7)	CRC (N=187)	Higher OS	(248)
CT (FOLFIRI) + targeted therapy: (bevacizumab)	*CCL2* rs4586 (chr.17)	Metastatic CRC (N=424)	Higher PFS	(249)
CT anthracyclines	*TLR4* rs4986790 (chr.9) and rs4986791 (chr.9)	Breast cancer (N=47 and 111)	Poor efficacy and early relapse	(250)
RT (60 Gy) + CT (Temozolomide)	*CCR2* rs1799864 (chr.9) or *PTENP1* rs7853346 (chr.9) via lncRNA-PTENP1/miR-19b/CCR2 pathway	Glioma (N=279)	Alleviated cognitive impairment after treatment	(251)
RT (≧64.8 Gy)	*CCL2* rs1024611(chr.17)	Nasopharyngeal carcinoma (N=411)	AA and AG genotypes correlate with distant metastasis	(252)
IT: PD-1/PD-L1 checkpoint inhibitors	Signature of *CCL2* rs13900 (chr.17) and rs4586 (chr.17); *NOS3* rs1799983 (chr.7); *IL6R* rs4845618 (chr.1); *IL12B* rs3212227 (chr.5); *IL1RN* rs419598 (chr.5); *CXCR3* rs2280964 (chr.X)	NSCLC, Renal cell carcinoma HNSCC Melanoma (n=94)	Good response to anti-PD-1/PD-L1 treatment	(253)

CRC, colorectal cancer; FOLFIR, folinic acid, fluorouracil and irinotecan; HNSCC, head and neck squamous cell carcinoma; NSCLC, non-small cell lung carcinoma; OS, overall survival; PFS, progression-free survival.

## Conclusion

Programming of monocyte progenitors in the bone marrow and in the circulation is essential for their potential to give rise to TAMs, DCs and MDSCs that are mostly cancer promoting, however, in some cancers (for example, in colorectal cancer) can have anti-cancer activities. Cancer therapy can interact with monocytes on the level of programming their dividing precursors in bone marrow, can affect efficiency of monocyte entry into the circulation, affect their subpopulational content and transcriptome, and can also affect their recruitment into tumor sites and their potential to develop cancer-specific TAMs, DCs and MDCSs.

However, the mechanisms that mediate cancer therapy programming of monocytes are only emerging, and large effort is needed to create a comprehensive picture for programming of major TAM, DC and MDSC precursors-monocytes in each type of cancer and by each type of therapy or their combination.

Rapidly developing single cell analytic technologies provide a highly useful tool here, since monocytes are much easier to examine on a single cell level compared to TAMs, DCs and MDSCs that need sophisticated approaches to be isolated out of heterogeneous tumor tissues without being damaged and without losing of highly adherent subpopulations. Moreover, in perspective, multiomics approaches have to be applied in order to combine the information not only about transcriptome and surface markers, but also about the metabolic status of monocytes, their genome and epigenome. Overall, the impact of genetic diversity of monocytes is underestimated when we examine the mechanisms of innate immune control in cancer. Such complex analysis is needed to design monocyte-targeted therapies, that due to the minimal invasiveness is a very promising direction to instruct human innate immunity to fight against cancer.

## Author contributions

Conceptualization: MP and JK. Writing—original draft preparation: MP, AF, IL, JK, SA, AT, NC. Writing— review and editing: MP, IL, JK. Figure preparation: AF, MP, JK. Supervision: NC and JK. Funding acquisition: JK. All authors contributed to the article and approved the submitted version.

## Funding

This research was funded state contract of the Ministry of Science and Higher Education of the Russian Federation “Genetic and epigenetic editing of tumor cells and microenvironment in order to block metastasis” o. 075-15-2021-1073.

## Conflict of interest

The authors declare that the research was conducted in the absence of any commercial or financial relationships that could be construed as a potential conflict of interest.

## Publisher’s note

All claims expressed in this article are solely those of the authors and do not necessarily represent those of their affiliated organizations, or those of the publisher, the editors and the reviewers. Any product that may be evaluated in this article, or claim that may be made by its manufacturer, is not guaranteed or endorsed by the publisher.
